# Large Domain Motions in Ago Protein Controlled by the Guide DNA-Strand Seed Region Determine the Ago-DNA-mRNA Complex Recognition Process

**DOI:** 10.1371/journal.pone.0054620

**Published:** 2013-01-29

**Authors:** Zhen Xia, Tien Huynh, Pengyu Ren, Ruhong Zhou

**Affiliations:** 1 Computational Biology Center, IBM Thomas J. Watson Research Center, Yorktown Heights, New York, New York, United States of America; 2 Department of Biomedical Engineering, The University of Texas at Austin, Austin, Texas, United States of America; 3 Department of Chemistry, Columbia University, New York, New York, United States of America; University of Leeds, United Kingdom

## Abstract

The recognition mechanism and cleavage activity of argonaute (Ago), miRNA, and mRNA complexes are the core processes to the small non-coding RNA world. The 5′ nucleation at the ‘seed’ region (position 2–8) of miRNA was believed to play a significant role in guiding the recognition of target mRNAs to the given miRNA family. In this paper, we have performed all-atom molecular dynamics simulations of the related and recently revealed Ago-DNA:mRNA ternary complexes to study the dynamics of the guide-target recognition and the effect of mutations by introducing “damaging” C·C mismatches at different positions in the seed region of the DNA-RNA duplex. Our simulations show that the A-form-like helix duplex gradually distorts as the number of seed mismatches increases and the complex can survive no more than two such mismatches. Severe distortions of the guide-target heteroduplex are observed in the ruinous 4-sites mismatch mutant, which give rise to a bending motion of the PAZ domain along the L1/L2 “hinge-like” connection segment, resulting in the opening of the nucleic-acid-binding channel. These long-range interactions between the seed region and PAZ domain, moderated by the L1/L2 segments, reveal the central role of the seed region in the guide-target strands recognition: it not only determines the guide-target heteroduplex’s nucleation and propagation, but also regulates the dynamic motions of Ago domains around the nucleic-acid-binding channel.

## Introduction

The essential role of small noncoding RNAs in the mechanisms of gene expression and regulation in eukaryotes has attracted a great deal of attention [1]–[12]. One of the small noncoding RNAs–microRNAs (miRNAs)–have been predicted to participate in almost all cellular processes and regulate more than 60% of all protein-coding genes in mammals [13]–[16]. Some Argonaute (Ago) family proteins, known as a critical component of RNA induced silencing complexes (RISC), can direct the degradation of the passenger strand of miRNA to form the RISC-loading complex with a guide strand (the mature miRNA) after Dicer cleavage, and then mediate the Ago-catalyzed mRNA cleavage by recognizing complementary target mRNA [17]–[20]. Therefore, the ability of miRNA and mRNA to complement each other is the key factor in determining the spectrum of regulatory targets for a given miRNA.

One major discovery is that the cleavage requires the Watson-Crick (WC) pairing at the 5′ region of the guide miRNA on nucleotide 2–8 (the ‘seed’ region) [15], [21]. Recent crystal structure of ternary complexes of eubacterial *Thermus thermophilus* Ago (*Tt*Ago) revealed an A-helical-like heteroduplex structure of the guide DNA (equivalent to miRNA) and target mRNA in the seed region [22], [23]. Follow-up studies of target RNAs with various lengths illustrated the pivot-like domain movement during the formation of guide–target duplex from nucleation to propagation steps [24]. Both single mismatch and pairwise 2′-O-methyl modification in the seed region of the guide strand have experimentally shown to have little influence on the cleavage activity [23]. However, the detailed dynamics and energetics (e.g. conformational changes, mRNA recognition dynamics, and interaction energy loss, etc) involved in these single- or multi-mismatches in the seed region are still far from completely understood. Thus, it will be interesting to see how these mismatches affect the guide-target strands’ nucleation and structure (as compared to the original A-helical-like duplex structure), as well as the Ago domain movements. It is also elusive how broad the target mRNAs can be recognized by a specific Ago-miRNA(DNA) family, which is crucial for designing de-novo mRNA-specific miRNA for therapies against diverse human diseases [25].

To address these above assumptions and questions, we have performed molecular dynamics (MD) simulations for both wild-type and the various mutants of the Ago ternary complexes (Ago protein bound with guide DNA strand and target mRNA) based on *Tt*Ago structures [23], [24]. *Tt*Ago is considered to be a good model to study the properties of Ago complexes because of its high structural and functional similarities to the eukaryotic Ago’s. We introduced damaging C·C mismatches (1 to 4 sites G to C mutations) at the seed region to find the maximum number of “tolerant mismatches” and also to study the molecular mechanism of the recognition process by gradually disrupting the Ago complex structure (which is analogous to protein unfolding simulations in order to reveal the folding mechanism). We further investigated the influence of 3′-compensatory pairing by using 15-bp guide-target duplex in Ago complexes with extended WC pairs at position 10–15. We also performed additional simulations, including using the latest version of CHARMM force field for RNA parameters (c36 parameter set) and a new Ago R172A mutation in L1/L2 “hinge-like” connection segment, to further validate our findings. Each MD simulation was performed for at least 100 ns to generate reasonably long trajectories to study the conformational changes and mRNA recognition dynamics, with a total aggregate MD simulation time of more than 1.7 µs. We observe large distortions of the guide-target heteroduplex in the ruinous 4-sites mismatch mutant, which give rise to a bending motion of the PAZ domain along the L1/L2 hinge and result in the opening of the nucleic-acid-binding channel. The long-range interactions between the seed region and PAZ domain reveal the central role of the seed region in the guide-target strands recognition. The seed region determines the guide-target heteroduplex’s nucleation and propagation, which regulates the dynamic motion of Ago domains and the mRNA recognition process.

## Systems and Methods

**Figure 1:**
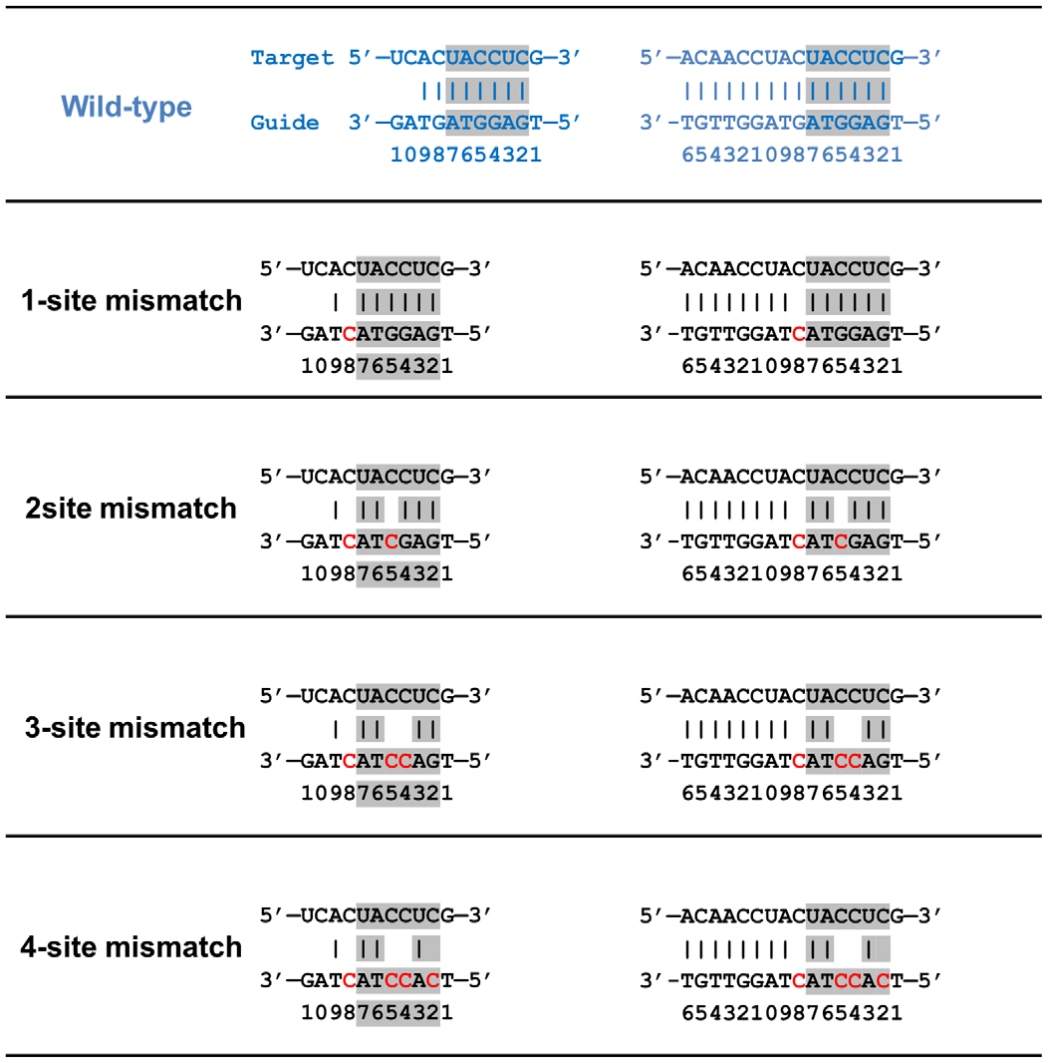
Sequences of the 11-bp and 15-bp DNA guides and mRNA target heteroduplex used in our simulations. Blue indicates the wild-type; mutated residues are shown in red, and residues spanning the seed region are shown in grey.

The x-ray crystal structure of wild-type *Tt*Ago bound to a 21-bp guide DNA and a 20-bp target mRNA complex (PDB entry: 3F73, released in December 2008) [23] was used as the starting structure for MD simulations, following our previous similar protocols [26]–[29]. We noticed that both DNA and mRNA strands can only be partly traced from position 1 to 11, and the base coordinates at position 10 and 11 are not available in the crystal structure. Therefore, the missing coordinates at position 10 and 11 were built from the known backbone structures, and the constructed complex (*Tt*Ago bound to 11-bp guide DNA-target mRNA) was then used in our following simulations. The disrupting C·C mismatches were introduced one by one to find the maximum “tolerant mismatches” at the seed region and also to reveal the potential molecular mechanism of the recognition process. These mutants include the 1-site mismatch mutant (on position 8), 2-site mismatch mutant (on position 5 and 8), 3-site mismatch mutant (on position 4, 5, and 8) and the 4-site mismatch mutant (on position 2, 4, 5, and 8) in the guide DNA (**Figure 1**). In order to evaluate the role of the L1/L2 “hinge-like” connection segment, an additional simulation was performed for arginine to alanine substitution at position 172 (R172A mutant) of Ago protein. All the Ago complexes (wild-type and mutants) were then solvated in ∼110×100×90 Å^3^ water boxes. A total of 32 Na^+^ ions and 29 Cl^-^ ions were added to neutralize and mimic the biological environment (100 mM NaCl concentration). The solvated systems contain approximately 100,400 atoms.

Following a similar procedure as described above, we have also repeated all the simulations for the newly released Ago complexes with a longer traceable guide-target duplex, with a length of 15-bp (position 2–16, PDB entry: 3HK2, released in October 2009) [24]. The same mutants with C·C mismatches have been applied to the 15-bp Ago ternary complexes as well (**Figure 1**). In the following discussion, we mainly focused on the simulation results from the Ago complexes with the 11-bp nucleic acid duplex, but both systems show comparable results.

The NAMD2 [30], [31] package was utilized for the MD simulations with the NPT ensemble. The CHARMM (parameter set c32b1) force field was used for the proteins and nucleic acids [32]–[36], and the TIP3P water was used as the explicit solvent [37], [38]. The Particle Mesh Ewald (PME) method [39], [40] was applied to treat the long-range electrostatic interactions and a 12 Å cutoff was employed for the van der Waals interactions. All the Ago complexes systems were equilibrated via a 20,000-step energy minimization, followed by 1-ns NPT MD equilibrations with 0.5 fs time step at 1 atm and 310 K. The equilibrated configurations were then used for the 100+ ns production runs. The time step for all production runs was 1.5 fs.

A newer version of the CHARMM force field for RNA parameters, which fixes the too much base-pair opening [41], was brought to our attention after we finished aforementioned simulations. To further validate our findings, we have repeated our simulations for the wild-type as well as the 4-site mismatch mutant with this latest RNA parameter set (CHARMM c36 parameter set, downloaded from http://mackerell.umaryland.edu/CHARMM_ff_params.html). Three independent runs were performed for both the wild-type and 4-site mismatch mutant for at least 100 ns each. Therefore, we have performed a total of 17 different MD simulations for the wild-type Ago-DNA-mRNA complex and its mutants with both the 11- and 15-bp guide-target duplex. The total aggregate MD simulation time is more than 1.7 µs.

## Results and Discussion

### Increasing Mismatches at the Seed Region causes Distortion of the A-form Helix

**Figure 2:**
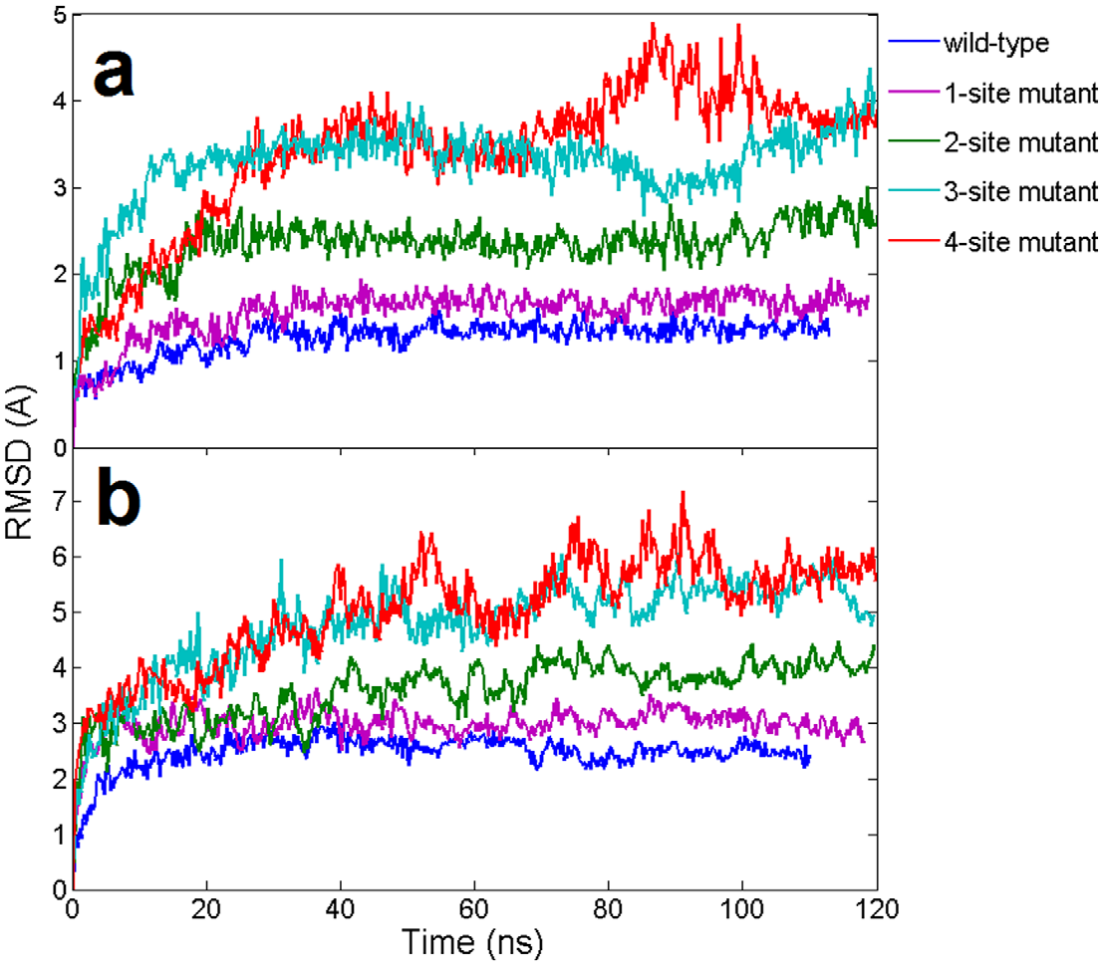
Time evolution of the backbone RMSDs of wild-type and mismatch mutants from their respective initial “native” structures using the 11-bp nucleic acid heteroduplex system. The results are obtained from 1 atm, 310 K NPT simulations (100∼120 ns). (**a**). RMSDs of the DNA-mRNA heteroduplex in Ago complexes (**b**). RMSDs of Ago protein in Ago complexes.

The interaction between Ago protein and the nucleic acid duplex are investigated by introducing ‘damaging’ G to C mutations at various positions in the guide DNA in the seed region. In this case, the G to C mutation will break the original G:C basepair and form C·C mismatches between the guide and the target strands. We designed 4 mutants whose number of C·C mismatches gradually increased from 1 to 4 (see Methods section and **Figure 1** for details). In our simulations, the overall backbone RMSDs of nucleic acid heteroduplexes showed that increasing mismatches in the seed region intensified the fluctuations of the DNA-RNA duplex (**Figure 2a**). For example, for the wild-type and the single mismatch mutant, the RMSDs reached a plateau around 1.5 Å and 1.8 Å after 20 ns, respectively (**Figure 2a**). These comparable results of wild-type and the 1-site mismatch mutant are consistent with the previous experimental study that single nucleotide mismatches at the seed region only slightly reduces the cleavage activity of Ago complexes [23]. On the contrary, the RMSDs for the 4-site mismatch mutants steadily increased to about 4 Å within ∼40 ns and then fluctuated at this elevated level with large amplitudes, indicating a potential detachment of the DNA-mRNA duplex. The RMSDs for the 3-site mismatch mutant also increased to ∼3Å within 15 ns. The RMDSs of 2-site mutant are in-between those of 1- and 3-site mutants, stable around 2∼3Å after a 20 ns MD simulation. Overall the increasing mismatches in the seed region undermined the stability of the DNA-mRNA heteroduplex.

**Figure 3:**
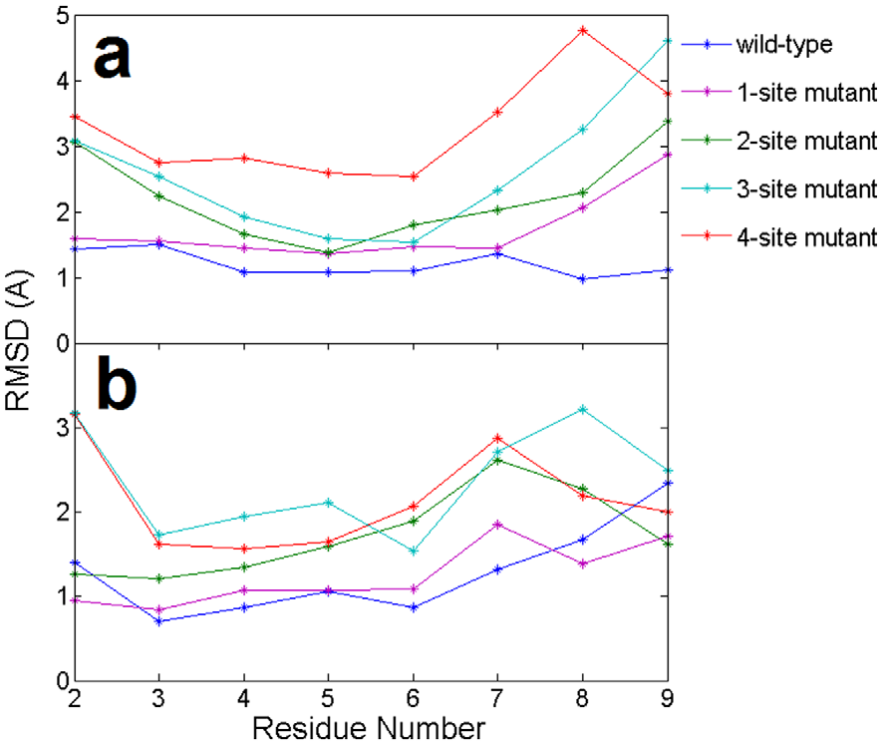
Comparison of the average RMSDs (per residue) in the (a) target mRNA and (b) guide DNA from the starting native structures of the wild-type and 4 mutants in the 11-bp nucleic acid heteroduplex. The results are obtained from 1 atm, 310 K NPT simulations (100∼120 ns). The X-axis indicates nucleotide position from the 5′ end of the guide DNA.

To assess the importance of the base pairing between DNA (miRNA) and the target mRNA, we calculated the average RMSD of each nucleotide’s backbone of the guide DNA and target mRNA (see **Figure 3**). The gradually raised RMSDs for each nucleotide corresponded well to the increased number of mutation sites, which indicates that mutations in the guide DNA affect not only their own corresponding base pairs, but also the entire guide-target duplex, even for the canonical WC basepairs. For example, basepairs at position 6 and 7 in the guide DNA were never mutated in all mutants, but we still saw larger average RMSDs in their corresponding target mRNA in the mutants compared to the wild-type, especially in the 4-site mutant (**Figure 3**). This is most likely because the 4-site mutant causes the overall “decoupling” of the DNA-mRNA duplex.

**Figure 4:**
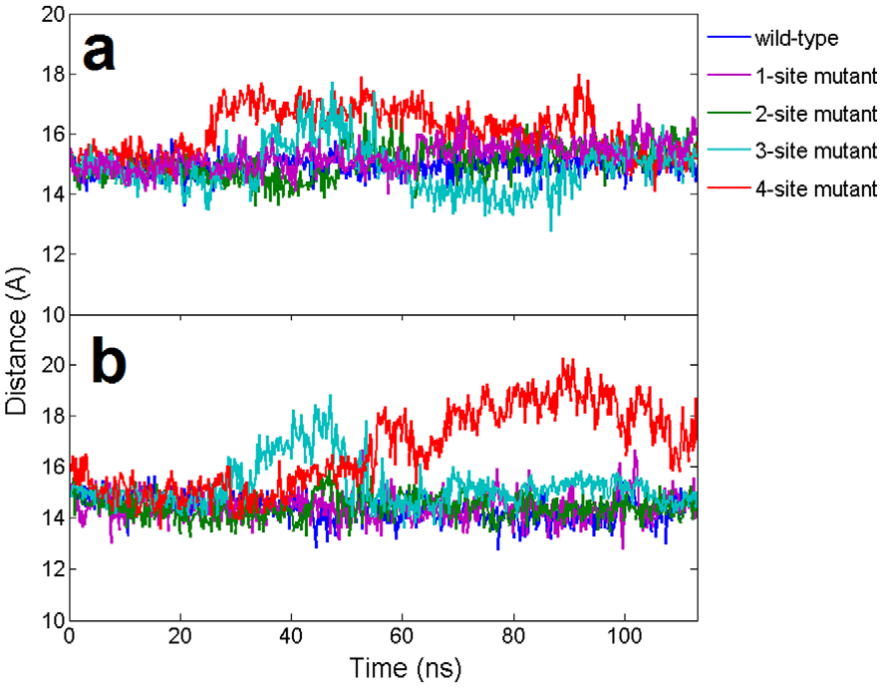
The dynamic distance of base pairs for the wild-type and 4 mutants in the 11-bp nucleic acid heteroduplex. Distances are calculated from the C4′–C4′ atoms between the guide DNA strand and the target mRNA strand at the same position. **(a)**. Distance of the A/T base pair at position 6 **(b)**. Distance of the U/A base pair at position 7.

**Figure 5:**
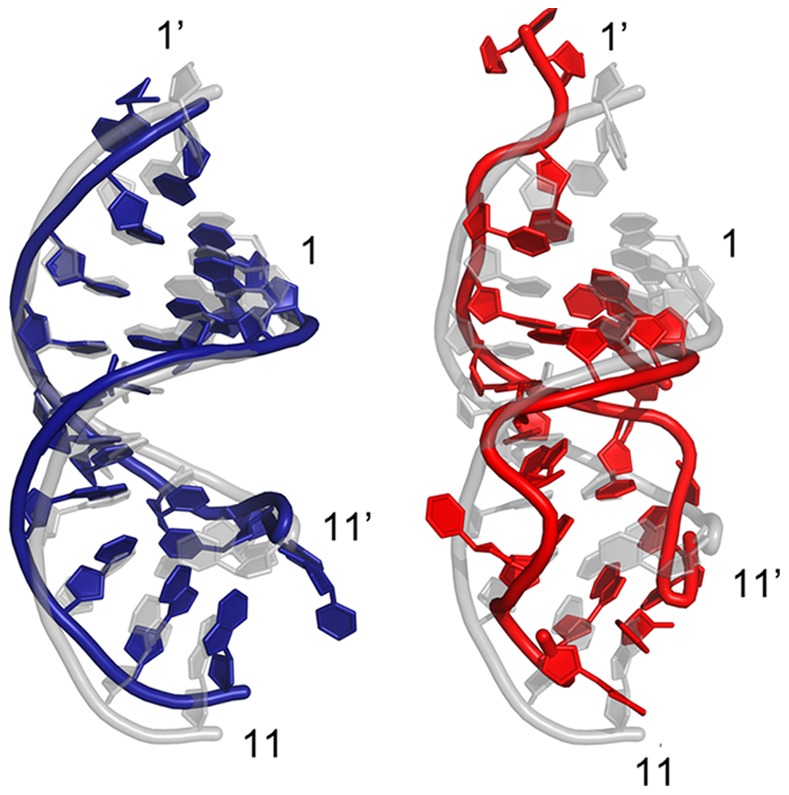
Structural comparison and distortion. Superposition of the final snapshot (colored in blue for the wild-type in the left panel and red for the 4-position mismatch mutant on the right) and the starting native structure (colored in light grey) for both the wild-type and the 4-site mismatch mutant of the 11-bp guide DNA/mRNA heteroduplex after 100+ ns of MD simulation. The backbone is represented as a tube and the rest are shown as plates. The numbers with prime (′) indicate that the nucleic acid belongs to the target strand. Larger distortion in the seed region (position 2 to 8) can be seen clearly on the 4-site mismatch mutant.

The stability of the seed region in mutants was further investigated by calculating the relative position of the backbone between two strands. Generally speaking, the C4′-C4′ distance should be ∼15 Å within each base pair for a typical A-form like duplex. In order to see whether the mutation could influence the stability of neighboring WC base pairs, we calculated the distance between wild-type base pairs at position 6 and 7 (see **Figure 4**). For wild-type and the single mismatch (1-site mutant), most of these C4′-C4′ pair distances remained intact at ∼15 Å, indicating a stable duplex structure. However, for the 3-site and 4-site mismatch mutants, the C4′-C4′ base pairs displayed significantly larger fluctuations, meaning that the A-form-like helix duplex was seriously distorted after 100 ns simulations. To confirm the conformational changes in atomic detail, we examined the dynamic structures of the guide-target duplex for all the trajectories and compared them with the final snapshots. The superposition of the final snapshot with its starting native structure for both the wild-type and the 4-site mutant are shown in **Figure 5**. During the 100-ns simulation, both the backbones and bases in the DNA-mRNA duplex for the wild-type were well kept, while for the 4-site mutant, most of the base pairs, including the canonical Watson-Crick ones, were disrupted during the 100 ns simulation. The severe backbone distortion in the guide-target duplex indicates the nucleation at the seed region cannot be formed in the 4-site mutant Ago complex. These findings also indicate the cumulative effect of these mismatches is non-additive. The seed region can tolerate 1 to 2 mismatches, but 3 or 4 mismatches (particularly the 4-site mutant), result in serious distortions and damages for the guide-target recognition.

The DNA-RNA heteroduplex was more likely to adopt a A-form helix in the crystal structure [23]. In our simulations, we found the A-form helix was well kept in the wild-type. The DNA-RNA heteroduplex started with a backbone RMSD of 1.61 Å and reached 1.82 Å at the end as compared to the standard A-form helix (**Figure S1a** and **S1b**). The structure deviations were much larger when compared to the standard B-form helix, with a backbone RMSD of 3.43 Å at beginning and 3.57 Å at the end of simulation (**Figure S1c** and **S1d**). These results of A-form-like conformation in our simulations are in excellent agreement with the previous studies on the structure of DNA-RNA hybrid duplex in RNase-H binding by Priyakumar and MacKerell [42]. We noticed that the A-form-like helix was gradually distorted as the number of mismatches increased at the seed. Neither A-form nor B-form helix was formed for the extreme 4-site mismatch mutant. It should be noted though that the guide DNA strand is half buried in the nucleic-acid-binding channel of the Ago protein, thus the conformation of the guide DNA strand might be less flexible than that in the free DNA-RNA hybrid duplex.

### Mismatches in the Seed Region Induces the Motion of PAZ Domain

**Figure 6:**
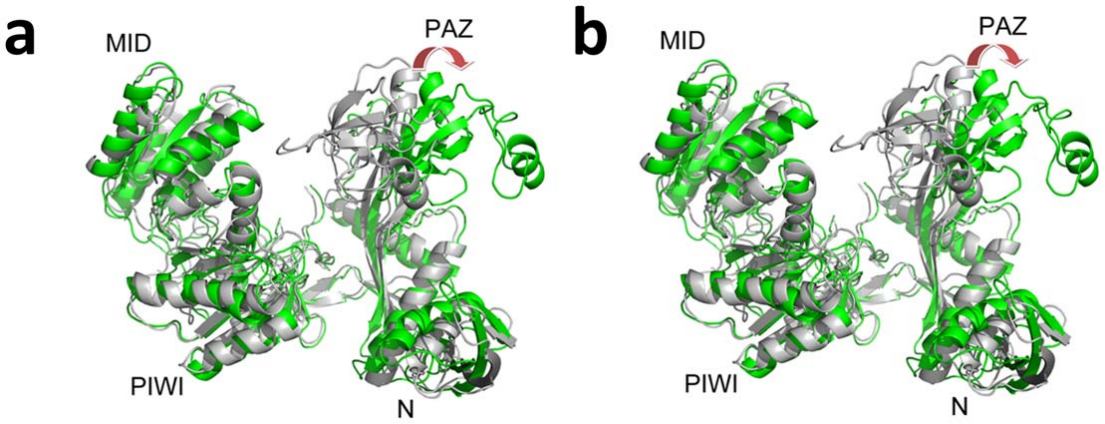
Structural view of the domain motions in the 4-site mutant Ago complexes with DNA as the guide strand. Two structures (one colored light grey and the other colored green) are picked from one 100-ns trajectory by principal component analysis and domain motion analysis. The 1^st^ principal component **(a)** and the 2^nd^ principal component **(b)** are shown here. The Ago protein is represented as cartoon and the four domain names are labeled. The red arrow here indicates the orientation of the domain motions.

A noticeable conformational difference was found between the binary Ago and ternary Ago complexes in their static X-ray crystal structures (**Figure S2**) [22], [23]. Structural alignments indicate that PAZ domain does display a large structural shift (opening) upon the binding with the DNA-RNA duplex (**Figure S2**). Large domain movements were also found in free Ago (Ago protein only) by previous normal model analysis and MD simulations [43], [44]. Here we focused on the ternary complex structural dynamics and the influence of the heteroduplex with mismatches in the seed. In our simulations, the overall RMSD of the Ago protein kept increasing for the 3-site and the 4-site mismatch mutants, up to ∼6Å within 100 ns, indicating some significant conformational changes or “unfolding” in part of the Ago protein due to the distortional force from the DNA-mRNA duplex (**Figure 2b**). To further investigate which domain or region in Ago proteins has led to the prominent fluctuation in mutants, we first performed Principal Component Analysis (PCA) for all the trajectories with the Gromacs package [45] and then put the 1^st^ and 2^nd^ principal components into the package DynDom [46], [47] to analyze the major domain motions (the domain motion analysis was carried out on website at http://fizz.cmp.uea.ac.uk/dyndom/). We found remarkable motions on PAZ domain for the 4-site mismatch mutant. The 1^st^ principal component showed the PAZ domain moved away from N domain (rotated and translated by 62.1° and 3.9 Å, respectively; see **Figure 6a**) and the 2^nd^ principal component showed that the major motion domain was PAZ again with further movement toward the PIWI-containing domain (rotated and translated by 47.3° and 4.3 Å, respectively; see **Figure 6b**). The superposition of the 4-site mismatch mutant final snapshot and the starting “native structure” clearly shows about a 25Å relative distance increase between the N domain and PAZ domain. On the other hand, we did not detect any significant domain motion for the wild-type Ago.

**Figure 7:**
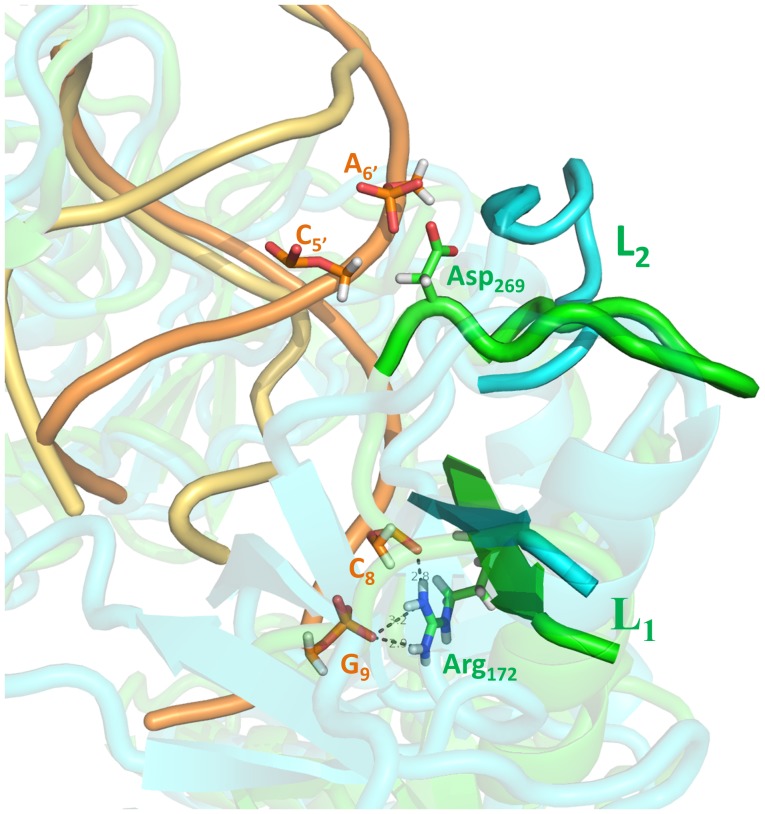
A closer view of the “hinge-like” bending at L1/L2 segment by superposing the final conformation and the starting native structure of the 4-site mutant with 11-bp guide DNA-target RNA duplex. The nucleic acid and L1/L2 segment in the wild-type are colored orange and green, respectively. The nucleic acid in the 4-site mutant is colored yellow and the L1/L2 segment is colored cyan. The residues that form hydrogen bonds between the guide-target duplex and L1/L2 segment are shown as sticks.

Interestingly, the native structure of Ago complexes shows no direct contact or interaction between the duplex seed region and the PAZ domain. Thus, one might be curious as to what “forces” have induced the PAZ domain motions in the 4-site mismatch mutant of Ago complexes. The domain motion analysis showed the bending residues responsible for the domain motion in Ago proteins were residues 168 to 176 and residues 270 to 279 in both the 1^st^ and 2^nd^ principal components. These two regions belong to the segments L1 and L2, which connect PAZ domain to other domains, acting as a “hinge” in determining the PAZ domain’s orientations. The relative positions and neighboring contacts between the “hinge” segments and the guide-target duplex in the 4-site mismatch mutant trajectory were investigated (see **Figure 7**). During the simulation, 3 hydrogen bonds, between the side chain of Arg172 in the L1 segment and the oxygen atoms in the phosphate group of C8 and G9 in the guide DNA, were broken due to the distortion of the double helix in the 4-site mismatch mutant (**Figure 7**), indicating Arg172 might play a significant role in Ago’s binding with the duplex. The strong distortion in the target mRNA and nucleotides 6 to 10 of the guide DNA made enough room for L1 and L2 coils to bend. The final snapshot in the trajectory showed the L1 and L2 segments rotated ∼30° and ∼90°, respectively. These rotations in the “hinge” region finally induced the entire motions of PAZ domain, which largely opened the nucleic-acid-binding channel between PAZ- and PIWI-containing lobes. These simulation results indicate that large scale PAZ domain motions are needed to close the Ago binding channel for function, while lack of that due to the large fluctuations and distortions of the DNA-RNA duplex with the seed mismatches, results in the failure of the binding channel closure and thus reduced cleavage activity.

As mentioned above, Arg172 in the L1 “hinge”-like segment seems to play a significant role in the complex binding, thus, will a mutation at position 172 alone be sufficient to cause the protein Ago to open up the PAZ domain in the ternary complex? We then designed an *in silico* mutation at position 172 (R172A) to remove those aforementioned direct hydrogen-bondings. Indeed, somewhat larger fluctuations were observed in both the DNA-RNA heteroduplex and the Ago protein (**Figure S3**) upon the R172A mutation, indicating the single mutation does help the PAZ domain opening. However, no large scale domain motions were observed in the Ago protein, and a relatively stable A-form-like DNA-RNA heteroduplex was formed with no mismatches (**Figure S4**). Therefore, we think the large domain motions observed in the 4-site mutant are mainly caused by the distortions of the duplex due to mismatches in the seed region, and the loss of the direct hydrogen-bondings between Ago’s L1 segment (Arg172) and the guide DNA also plays a role in the complex stability, though a relatively minor one. From the target mRNA recognition point of view, if there is a significant mismatch in the seed region, the large fluctuations in the duplex (and thus distortions) will cause difficulty for the Ago protein to close its binding channel and perform the cleavage function.

### Cleavage Mechanism Revealed by the Long-range Interaction between the Seed Region and Ago Domains

The domain motions play an important role in the cleavage activity of Ago protein. Previous crystal structural studies presented an opening of nucleic-acid-binding channel from the binary structures to the ternary complexes (**Figure S2**) [23]. Additional conformational changes were also observed by increasing the length of target RNA [24]. Meanwhile, the 5′ nucleation at the seed region was believed to be the determinant in guiding the recognition of target mRNAs. Our current simulations strongly indicate that those domain motions are highly correlated to the structure stability of the seed region in the nucleic acid duplex, which consequently bridges the Ago cleavage activity and the heteroduplex nucleation of the seed. Our simulations show that the number of mismatches in the seed region determines the level of structure fluctuations and controls the binding affinities between the guide-target duplexes. If the seed region is completely matched or has only one C·C mismatch between the guide DNA and the target mRNA, the two strands could form a stable A-form-like helix after the nucleation. As the ternary structure has revealed [23], [24], the hydrogen bonds and other local contacts between nucleic acid and Ago protein induce the target mRNA to be captured tightly by the binary Ago complex. The L1/L2 “hinge”-like segment help orient the PAZ domain close to the N and PIWI domains. Thus, the nucleic acid duplex and the domains in Ago protein are all well-aligned in their catalytic positions, ready for the cleavage. Our discovery is consistent with previous cleavage activity studies with single nucleotide mismatches at each position in the guide strand, where weaker but still active cleavage reactions have been observed experimentally [23]. On the other hand, if more mismatches (3 or 4) are found in the seed region between the guide DNA and the target mRNA, the large fluctuations and weak binding affinities may keep the nucleic-acid-binding channel open such that cleavage cannot be accomplished. Our simulations indicate such high fluctuations can push the improper target mRNA away from the Ago-DNA complex and leave space for the complementary target mRNA segment to bind.

Interestingly, the seed-mismatches induced large domain motions were also observed in miRNA guided mRNA cleavage in our previous molecular dynamics simulations on Ago-miRNA:mRNA complexes [48]. In a similar extreme 4-site C·C mismatch mutant, we observed striking bending motion of the PAZ domain along the L1/L2 ‘hinge’-link segment and a subsequent opening of the nucleic-acid-binding channel. Because of the higher flexibility nature of RNA comparing to DNA, the Ago-miRNA:mRNA ternary complexes can readily admit a wide variety of modifications, such as combinations of multiple G:U wobbles in the seed [49]–[51] (and interested readers can refer to Ref 48 and references therein).

### Similar Trends for Ago Complexes with Additional 3′-compensatory Pairing

**Figure 8:**
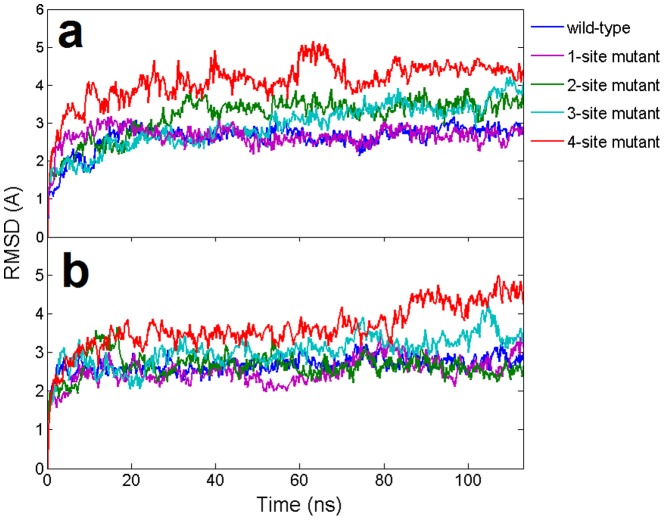
Time evolution of the wild-type backbone RMSDs and the mismatch mutants from their respective initial “native” structures using longer 15-bp nucleic acid heteroduplex system. The results are obtained from 1 atm, 310 K NPT simulations for 100∼120 ns. (**a**). The RMSDs of the DNA-mRNA heteroduplex in Ago complexes (**b**). The RMSDs of Ago protein in Ago complexes.

**Figure 9:**
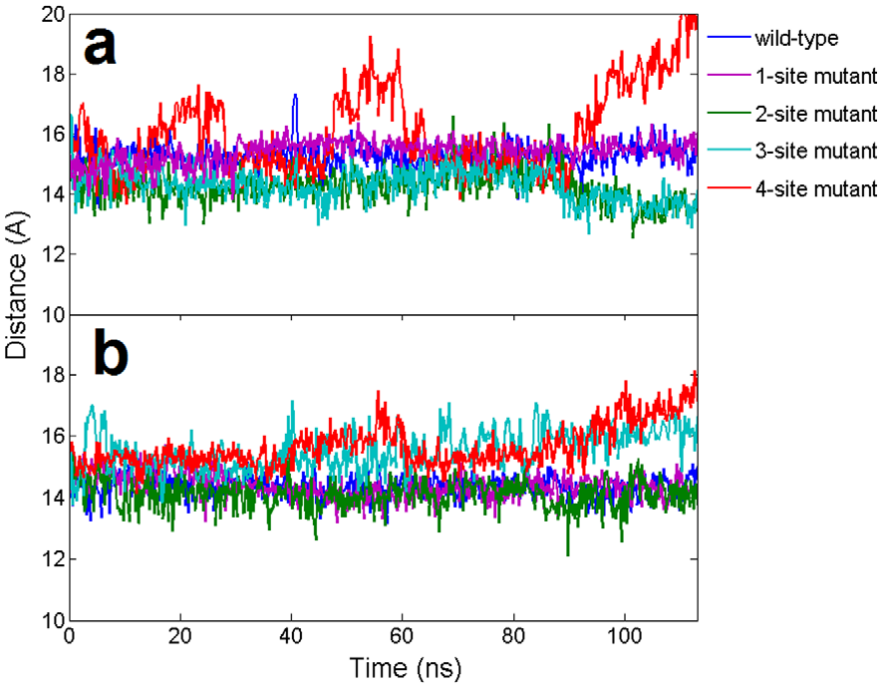
The dynamic distance of base pairs for the wild-type and 4 mutants in the longer 15-bp nucleic acid heteroduplex. Distances are calculated from the C4′–C4′ atoms between the guide DNA strand and the target mRNA strand at the same position. **(a)**. Distance of the A/T base pair at position 6 **(b)**. Distance of the U/A base pair at position 7.

**Figure 10:**
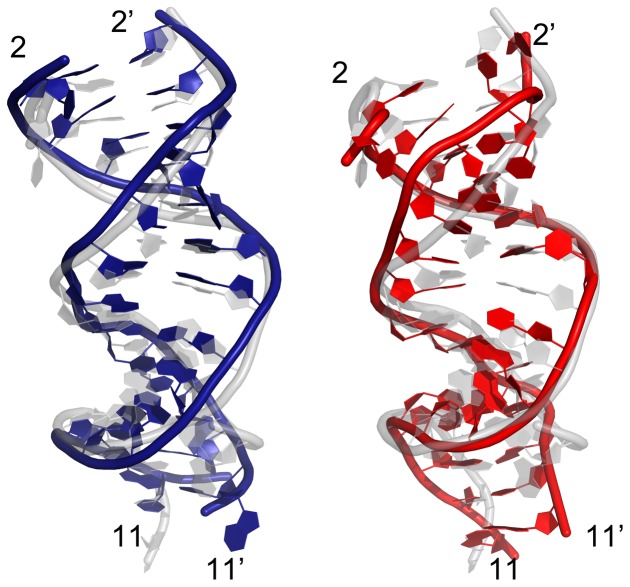
Structural comparison and distortion. Superposition of the final conformation (colored in blue for the wild-type on the left, and red for the 4-position mismatch mutant on the right) and the starting native structure (colored in light grey) for both the wild-type and the 4-site mismatch mutant using the longer 15-bp guide DNA/mRNA heteroduplex after ∼100 ns of MD simulation. The backbone is represented as a tube and the rest are shown as plates. The numbers with prime (′) indicate that the nucleic acid belongs to the target strand. Larger distortion in the seed region (position 2 to 8) can be clearly seen on the 4-site mismatch mutant.

Except for the seed region, the 3′-compensatory pairing are thought to offset the seed mismatches and enhance the binding specificity and affinity between the guide and target strands [15], [52]. However, the lack of experimental evidence has left this assumption an open question so far [15]. In order to investigate whether there is a significant difference between the longer target mRNA duplex (15-bp) and the 11-bp duplex systems, we performed similar molecular dynamics simulations for the Ago complexes with additional 3′-compensatory pairing from position 10 to 15. Both the wild-type and corresponding four different mismatch mutants at the seed region were simulated (**Figure 1**). Overall, similar trends with somewhat smaller fluctuations as compared to the 11-bp system were observed in RMSDs of Ago protein and the guide-target duplex from their native structures during simulations (**Figure 8**). Larger fluctuations, again, were observed in the 3- and 4-site mismatch mutants in the 15-bp complexes. The significant distortions in the seed region with 3- and 4-site mismatches were observed even with the additional propagated base pairs from positions 10 to 15 (**Figure 9** and **Figure 10**). Overall, our simulations results showed similar trends for Ago complexes with additional 3′-compensatory pairing and the conclusions from the 11-bp duplex complexes should be applicable for the 15-bp ones.

### Further Validation with the Latest Version of RNA Force Field Parameters

A new version of RNA parameters in CHARMM force field is available very recently after we performed all the aforementioned simulations [41]. The original problem of too much Watson-Crick basepair opening was corrected in this latest CHARMM force field (parameter set c36). In order to further validate our current findings, we repeated our MD simulations for the wild-type and the 4-site mismatch mutant using the latest RNA parameters (see Method section for more simulation details). We found very similar results as those from the original force field (c32b1 parameter set), where again small RMSDs (1.5 Å) and stable A-form heteroduplex were seen in the wild-type, but larger fluctuations (RMSD>3 Å) and significant seed distortions were observed in the 4-site mismatch mutant (Figure S5).

### Conclusion

We have performed a series of molecular dynamics simulations on Ago-DNA:mRNA ternary complexes to investigate the influence of multi-mismatches on Ago domain motions and its cleavage activities. The systems we studied include the wild-type and four different mismatch mutants in the seed region of the guide strand. We found that an increasing number of mismatches in the seed region gradually induced more intense fluctuations in the guide-target duplex at the nucleic-acid-binding channel of Ago protein. Similar trends with slightly more stable structures were found for duplexes with additional 3′-compensatory pairing.

The extreme 4-stie mismatch mutant not only distorted the A-form-like helix duplex, but also induced large conformational changes in L1 and L2 segments of the Ago protein. The PCA and domain motion analysis showed a significant motion in PAZ domain mediated by a “hinge”-like bending in L1 and L2 segments. The PAZ domain motions induced by the long-range interactions (or fluctuations) from the distorted seed region (in analogues to the protein unfolding simulations) revealed a potential molecular mechanism of how the Ago complexes recognize the target mRNA. We found that the number of mismatches in the seed region determines the fluctuation level of the guide-target duplex, and the fluctuation level of the guide-target duplex then influences the conformations of the L1/L2 “hinge”-like segments and the PAZ domain. The PAZ domain motion is an essential component of the entire pivot-like domain movements in Ago scaffold [22]–[24], [43]. The conformational dynamics in the seed region not only determines the guide-target duplex’s nucleation and propagation, but also regulates the domain motions in the Ago protein. These simulation results indicate that large scale PAZ domain motions are needed to close the Ago binding channel for function, while lack of that due to the large fluctuations and distortions of the DNA-RNA duplex with the seed mismatches, results in the failure of the binding channel closure and thus reduced cleavage activity.

These atomistic-level molecular dynamics simulations might have shed light to better understanding of the structure and function of non-conserved seed region in Ago complexes. The important role of the seed region may be more complicated and diverse than simple sequence complementarities, as indicated in our recent studies on Ago-miRNA:mRNA complexes, which show a large repertoire of admissible “seed-less” targets [48]. The association of Ago domain motions and the seed mismatches through long-range interactions may provide a novel way to study the cleavage mechanism.

## Supporting Information

Figure S1 **Superposition of the seed region (position 2–8) of DNA-RNA heteroduplex to the standard A-form (a and b, colored in light gray) and B-form helix (c and d, colored in light gray) in the wild-type Ago complex simulation.** The starting structures are colored in green and the final snapshots are colored in blue. The backbone is represented as a tube and the rest are shown as plates. The numbers with prime (´) indicate that the nucleic acid belongs to the target strand.
(TIF)

Figure S2 **Superposition of binary Ago protein (colored in yellow) and ternary Ago protein (colored in green).** The Ago proteins are shown in cartoon. Structural alignments indicate that PAZ domain does display a large structural opening upon the binding with the DNA-RNA duplex.
(TIF)

Figure S3 **Time evolution of the backbone RMSDs of the R172A mutant from the starting structure.** The results are obtained from 1 atm, 310 K NPT simulations (100 ns).
(TIF)

Figure S4 **Structural changes upon the R172A mutation.** The final snapshot (colored in cyan and green) are superposed to the starting structures (colored in light grey) after 100 ns of MD simulation. (**a**) is the Ago protein, and (**b**) shows the DNA-RNA duplex. The L1, L2 segments are highlighted in magenta and the R172A mutation site is shown as sphere. The backbones are represented as cartoon and the bases are shown as plates. The numbers with prime (´) indicate that the nucleic acid belongs to the target strand.
(TIF)

Figure S5 **Time evolution of the backbone RMSDs of the wild-type and 4-site mismatch mutants from their starting structures.** These simulations were performed with new CHARMM force field parameters (set C36) for RNA. **(a)**. RMSDs of the DNA-mRNA heteroduplex in Ago complexes; **(b)**. Superposition of the final snapshot (colored in blue for the wild-type in the left panel and red for the 4-position mismatch mutant in the right) and the starting native structure (colored in light grey) for both the wild-type and the 4-site mismatch mutant. The backbones are represented as tube and the rest are shown as plates. The numbers with prime (′) indicate that the nucleic acid belongs to the target strand.
(TIF)
