# Correction: Large Domain Motions in Ago Protein Controlled by the Guide DNA-Strand Seed Region Determine the Ago-DNA-mRNA Complex Recognition Process

**DOI:** 10.1371/annotation/dc754ef5-000d-4362-be14-e8b04d5e77e1

**Published:** 2013-08-08

**Authors:** Zhen Xia, Tien Huynh, Pengyu Ren, Ruhong Zhou

A grant to the first and third authors was incorrectly omitted from the Funding Statement. The following sentence should be read following the first sentence in the Funding Statement: "Z.X. and P.R. are also supported by Robert A. Welch Foundation (F-1691)." 

