# Supplementary figures and images for: Large Domain Motions in Ago Protein Controlled by the Guide DNA-Strand Seed Region Determine the Ago-DNA-mRNA Complex Recognition Process
Source: PLoS One. 2013 Jan 29;8(1):e54620. doi: 10.1371/journal.pone.0054620 (PMC3558513; doi:10.1371/journal.pone.0054620)

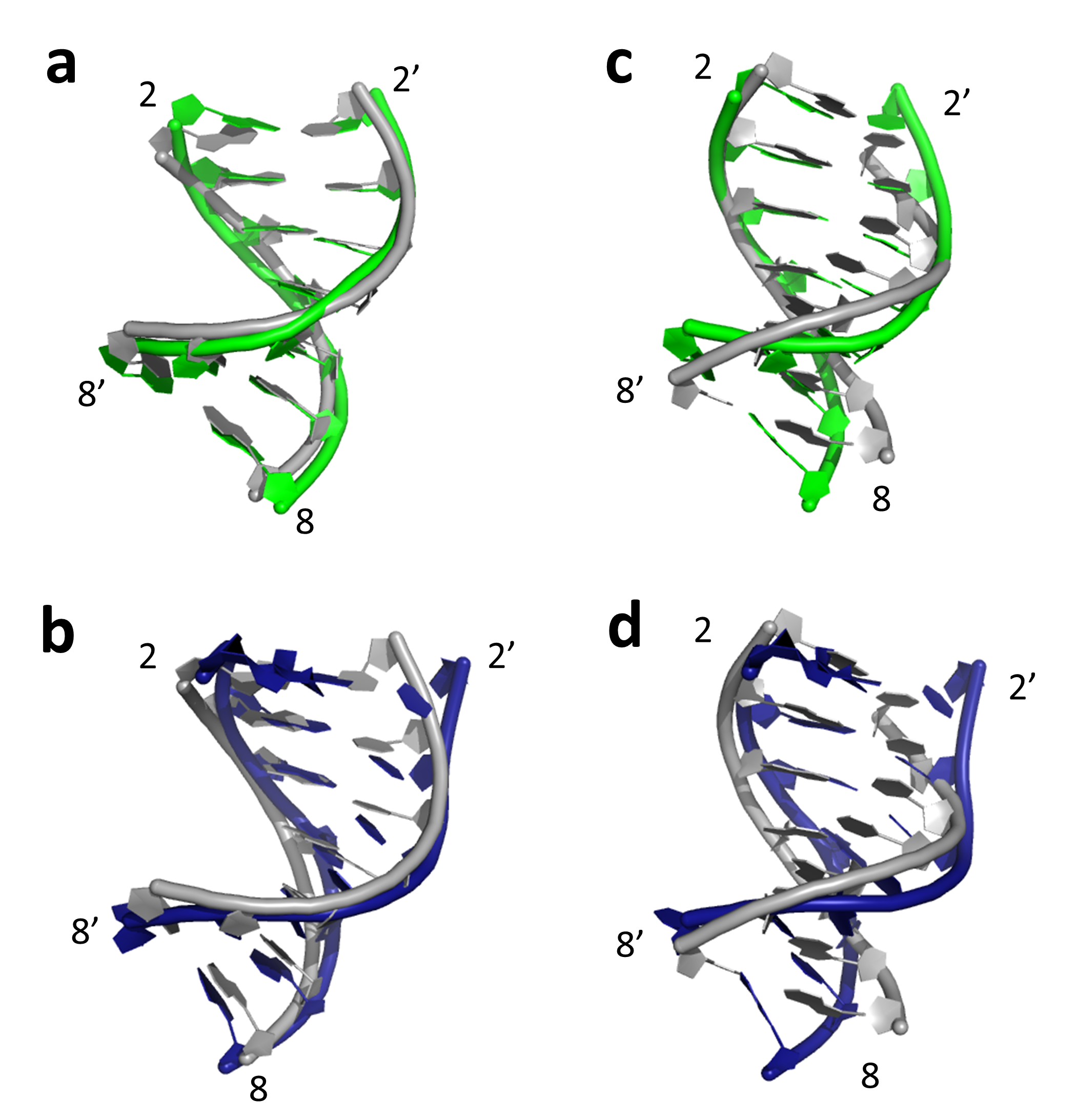

Supplement: Figure S1 — Superposition of the seed region (position 2–8) of DNA-RNA heteroduplex to the standard A-form (a and b, colored in light gray) and B-form helix (c and d, colored in light gray) in the wild-type Ago complex simulation. The starting structures are colored in green and the final snapshots are colored in blue. The backbone is represented as a tube and the rest are shown as plates. The numbers with prime (´) indicate that the nucleic acid belongs to the target strand. (TIF) [file pone.0054620.s001.tif]

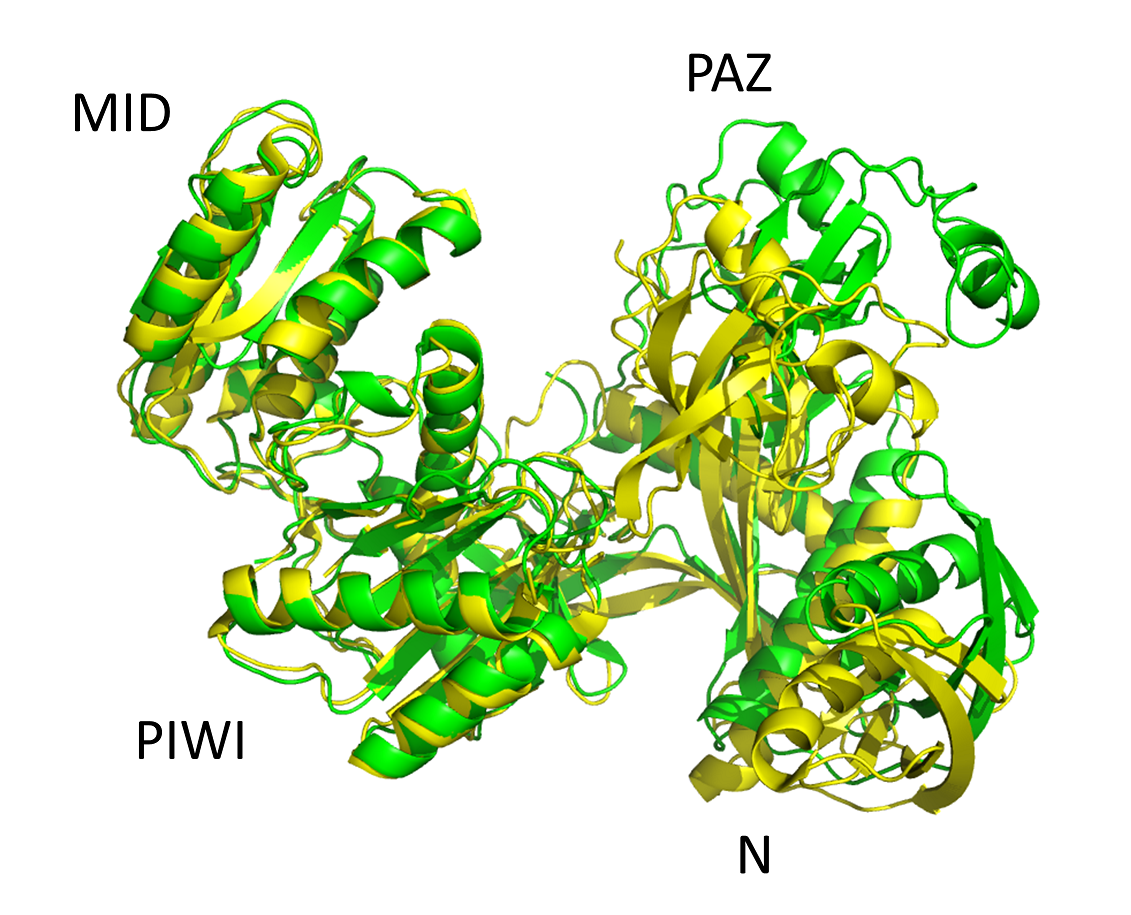

Supplement: Figure S2 — Superposition of binary Ago protein (colored in yellow) and ternary Ago protein (colored in green). The Ago proteins are shown in cartoon. Structural alignments indicate that PAZ domain does display a large structural opening upon the binding with the DNA-RNA duplex. (TIF) [file pone.0054620.s002.tif]

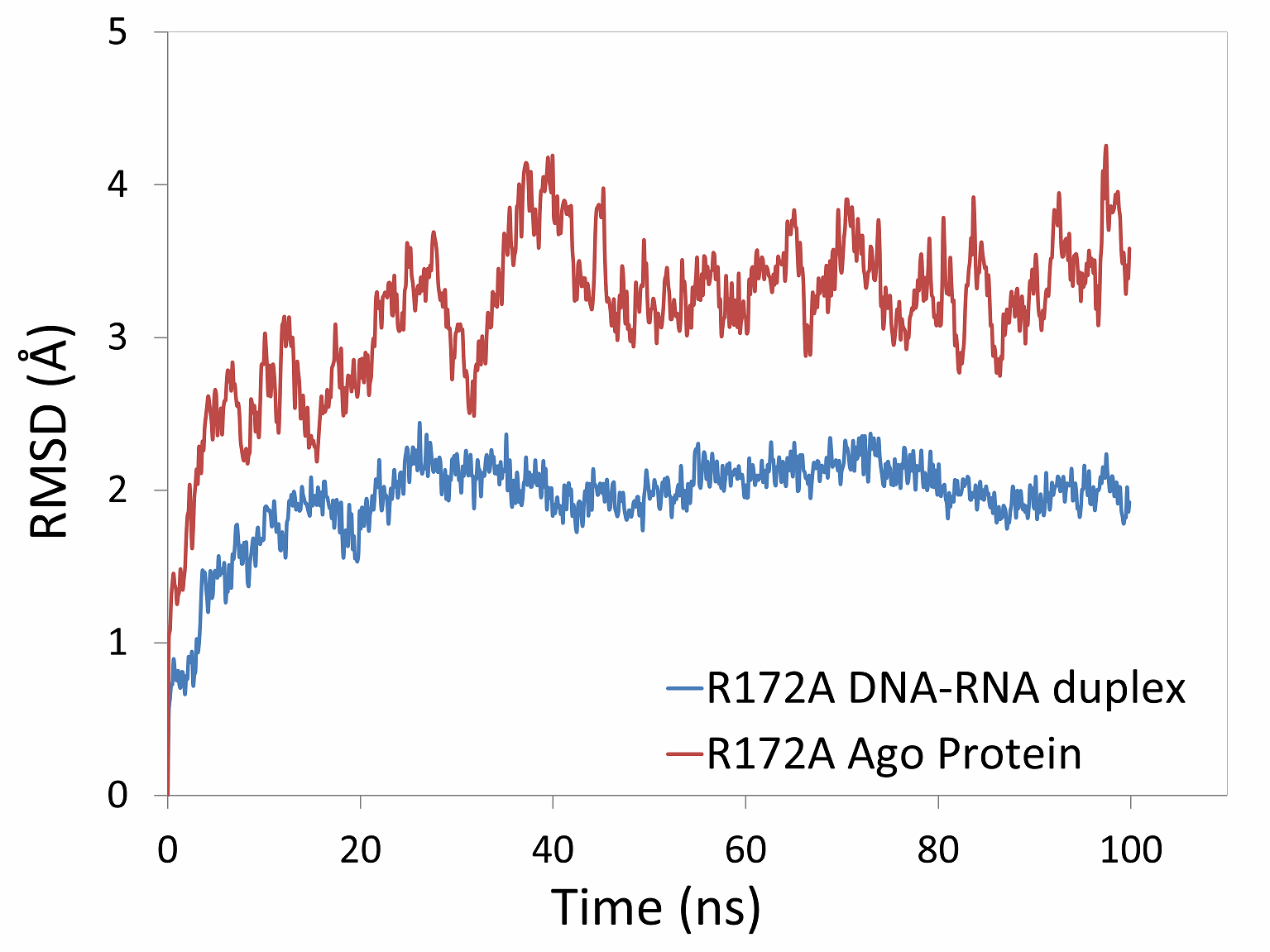

Supplement: Figure S3 — Time evolution of the backbone RMSDs of the R172A mutant from the starting structure. The results are obtained from 1 atm, 310 K NPT simulations (100 ns). (TIF) [file pone.0054620.s003.tif]

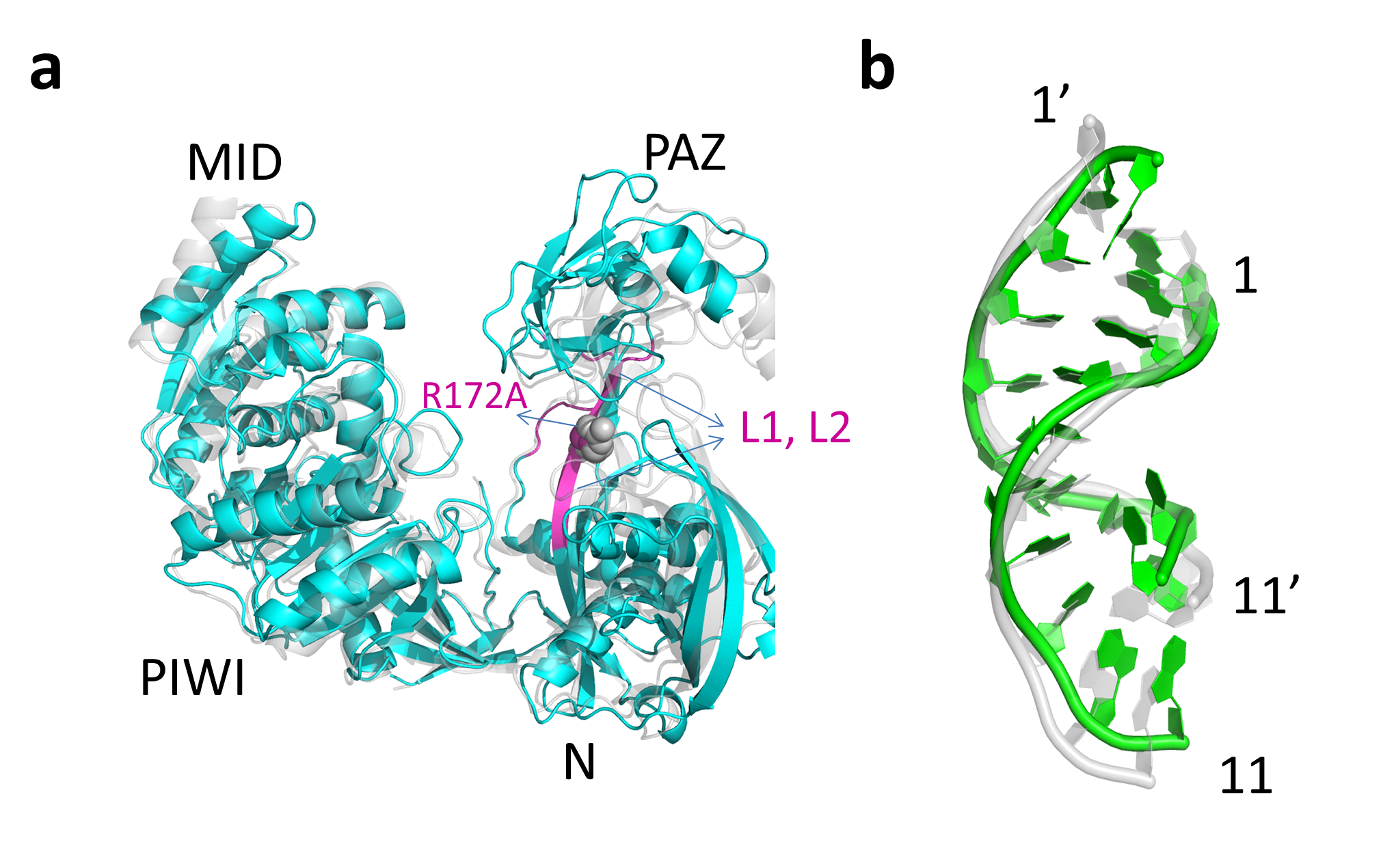

Supplement: Figure S4 — Structural changes upon the R172A mutation. The final snapshot (colored in cyan and green) are superposed to the starting structures (colored in light grey) after 100 ns of MD simulation. (a) is the Ago protein, and (b) shows the DNA-RNA duplex. The L1, L2 segments are highlighted in magenta and the R172A mutation site is shown as sphere. The backbones are represented as cartoon and the bases are shown as plates. The numbers with prime (´) indicate that the nucleic acid belongs to the target strand. (TIF) [file pone.0054620.s004.tif]

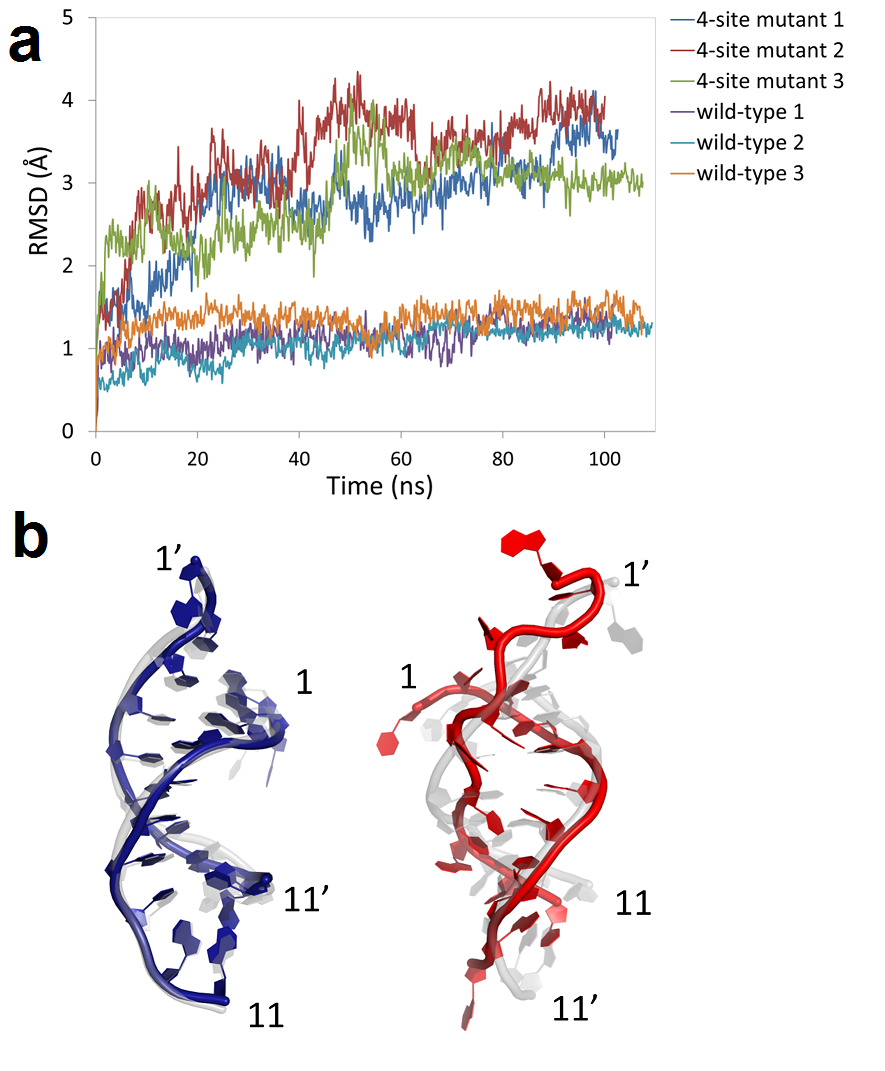

Supplement: Figure S5 — Time evolution of the backbone RMSDs of the wild-type and 4-site mismatch mutants from their starting structures. These simulations were performed with new CHARMM force field parameters (set C36) for RNA. (a). RMSDs of the DNA-mRNA heteroduplex in Ago complexes; (b). Superposition of the final snapshot (colored in blue for the wild-type in the left panel and red for the 4-position mismatch mutant in the right) and the starting native structure (colored in light grey) for both the wild-type and the 4-site mismatch mutant. The backbones are represented as tube and the rest are shown as plates. The numbers with prime (′) indicate that the nucleic acid belongs to the target strand. (TIF) [file pone.0054620.s005.tif]
